# Longitudinal changes in functional capacity in frontotemporal dementia and Alzheimer's disease

**DOI:** 10.1002/dad2.70028

**Published:** 2024-11-15

**Authors:** David Foxe, Muireann Irish, Sau Chi Cheung, Mirelle D'Mello, Yun Tae Hwang, James Muggleton, Nicholas J. Cordato, Olivier Piguet

**Affiliations:** ^1^ The University of Sydney School of Psychology Sydney Australia; ^2^ The University of Sydney Brain and Mind Centre Sydney Australia; ^3^ Royal Prince Alfred Hospital Neuropsychology Unit Sydney Australia; ^4^ University of Newcastle Central Coast Clinical School Newcastle Australia; ^5^ Gosford Hospital Department of Neurology Gosford Australia; ^6^ Prince of Wales Hospital Department of Aged Care Sydney Australia; ^7^ University of New South Wales St George Clinical School Sydney Australia; ^8^ St George Hospital The Department of Aged Care Sydney Australia; ^9^ Calvary Community Health Calvary Health Care Kogarah Sydney Australia

**Keywords:** activities of daily living, Alzheimer's disease, frontotemporal dementia, longitudinal assessment, primary progressive aphasia

## Abstract

**INTRODUCTION:**

This study investigated the changes in functional capacity with disease progression in a well‐characterised cohort of patients diagnosed with frontotemporal dementia (FTD) and Alzheimer's disease (AD) presentations.

**METHODS:**

We recruited 126 behavioural variant FTD (bvFTD), 40 progressive nonfluent aphasia (PNFA), 64 semantic dementia (SD), 45 logopenic progressive aphasia (LPA), and 115 AD patients. Functional capacity was measured annually over ∼7 years using the Disability Assessment for Dementia.

**RESULTS:**

Linear mixed effects models revealed the bvFTD group demonstrated disproportionate functional impairment at baseline and over the study period. Functional capacity among the other syndromes showed a more uniform pattern of decline, with less severe functional impairment at baseline and ∼7%–10% mean annual decline. Baseline correlations indicated different mechanisms supporting basic and complex functional proficiency among the groups.

**DISCUSSION:**

Our findings demonstrate distinct functional profiles across dementia syndromes with disease progression. Identifying progression milestones across syndromes will improve clinical management.

**Highlights:**

- bvFTD shows severe functional impairment at baseline and over time.
- PNFA, SD, LPA, AD: less severe baseline functional impairment; more uniform decline.
- General cognition is related to IADLs, but not BADLs, in all groups.
- Behavioural disturbances relate to IADLs and BADLs in bvFTD and SD.
- Behavioural‐ADL relations are more mixed in PNFA, LPA, and AD.

## 1 INTRODUCTION

Clinical diagnostic criteria for frontotemporal dementia (FTD) and Alzheimer's disease (AD) have long been established.^1^, ^2^, ^3^ These criteria focus primarily on the constellations of cognitive and behavioural changes most commonly found in these syndromes: behaviour and personality in behavioural variant frontotemporal dementia (bvFTD), language in semantic dementia (SD), and progressive nonfluent aphasia (PNFA), and memory and language in the amnestic and language presentations of AD.

Although not necessarily part of the core diagnostic criteria for FTD and AD syndromes, other disturbances beyond cognition and behaviour are commonly observed. Indeed, functional impairments, that is, difficulties completing tasks or actions essential for independent living, are prominent features in dementia.^4^, ^5^, ^6^, ^7^, ^8^, ^9^, ^10^, ^11^, ^12^, ^13^ The integrity of functional capacity is typically assessed by evaluating an individual's ability to successfully complete common day‐to‐day activities of varying complexity.^14^ These activities are categorised into basic (BADL) or instrumental (IADL) activities of daily living. BADL encompasses activities such as personal care, dressing, and eating, which require minimal cognitive effort. In contrast, IADL involves activities with higher cognitive demands, such as meal preparation, shopping, home maintenance, and financial management. Disturbances in IADL often emerge in the early stages of the disease, while impairments in BADL tend to manifest in more advanced stages.^4^, ^5^, ^9^

Functional capacity varies widely across dementia syndromes.^5^, ^7^, ^11^ Early functional disturbances have been reported in bvFTD,^4^, ^5^, ^6^, ^13^, ^15^ but less so in the other FTD syndromes or the amnestic and non‐amnestic (e.g., logopenic progressive aphasia; LPA) presentations of AD.^4^, ^5^, ^6^, ^7^, ^8^, ^9^, ^10^, ^13^, ^15^ Understanding of the evolution of functional impairment over time in these different dementia syndromes, however, remains limited due to the lack of comprehensive longitudinal studies comprising multiple time points. In addition, these functional impairments tend to co‐occur with changes in cognition and behaviour, although their relations at baseline clinical presentation are not entirely well understood. For example, in the early stages of bvFTD, functional disturbances often co‐occur with prominent behavioural alterations (e.g., disinhibition, apathy), while cognition remains comparatively less affected.^5^, ^13^, ^16^, ^17^, ^18^, ^19^ In the language presentations of dementia (i.e., PNFA, SD, and LPA; collectively referred to as primary progressive aphasia [PPA]), functional disturbances appear primarily linked to the canonical speech and language difficulties, at least in the earlier disease stages.^20^, ^21^ Collectively, these findings suggest that the clinical trajectories of these dementia syndromes are more complex than previously thought,^22^, ^23^ highlighting a significant knowledge gap that has implications for disease characterisation, prognosis, and management.

**RESEARCH IN CONTEXT**

1. **Systematic review**: We reviewed the literature on functional decline in frontotemporal dementia (FTD) and Alzheimer's disease (AD) using Google Scholar and PubMed.
2. **Interpretation**: Our study showed that FTD and AD exhibit various functional capacity profiles at baseline and over time, adding to the growing literature. The behavioural variant of FTD (bvFTD) showed severe functional impairment at baseline and over time compared to other FTD and AD syndromes. At baseline assessment, general cognition related to instrumental activities of daily living (IADLs) but not basic ADLs (BADLs) across all groups. Behavioural disturbances related to IADLs and BADLs in bvFTD and semantic dementia, with mixed relations in other dementia syndromes.
3. **Future directions**: Our findings emphasise the need to identify key progression milestones across dementia syndromes, as this could improve clinical management and reduce carer burden. Interventions targeting IADLs should consider combining cognitive and behavioural approaches, while addressing BADLs may focus on behaviour.

Taken together, longitudinal studies examining the progression of functional impairments in FTD and AD syndromes, and their relation to cognition and behaviour at presentation, are limited and often lack comprehensive follow‐up data. To this end, the objectives of the current study were to (i) characterise changes in basic and complex functional capacity profiles in a large cohort of well‐characterised FTD/PPA and AD patients with variable disease severity, (ii) map the progression of these profiles over a 7‐year period, and (iii) identify the contributions of cognitive and behaviour deficits to these functional profiles.

## 2 METHODS

### 2.1 Patients

Three‐hundred‐and‐ninety individuals diagnosed with dementia by FRONTIER, the younger‐onset dementia research clinic at the Brain and Mind Centre, University of Sydney, Australia, between November 2007 and February 2020, were included in this study: 126 bvFTD, 115 AD, 64 SD (also referred to as semantic variant PPA), 45 LPA (also referred to as logopenic variant PPA), and 40 PNFA (also referred to as nonfluent variant PPA) (Table 1). All participants underwent a comprehensive neurological and neuropsychological assessment, and structural brain magnetic resonance imaging (MRI). Assessments were typically completed within a 1‐ to 2‐month period. Patients were reviewed at approximately 12‐month intervals. Diagnosis was considered at each clinical visit according to the relevant clinical diagnostic criteria at the time of testing (bvFTD^1^; PNFA, SD, LPA^2^; AD^3^). Carers completed clinical surveys and questionnaires annually, assessing the patient's functional ability and the presence of behaviour changes. These assessments were conducted either during visits to the research clinic or at home if the patient was no longer able to attend. Disease duration was calculated at the initial clinical visit based on the carer‐reported onset of symptoms.

**TABLE 1:** Demographic, cognitive, functional capacity, and behavioural profiles of the patient groups at baseline assessment.

Parameter	bvFTD (*n* = 126)	PNFA (*n* = 40)	SD (*n* = 64)	LPA (*n* = 45)	AD (*n* = 115)	Group Effect (F or H)	*p*	Post hoc test (Bonferroni corrected)
Sex (m: f)	80: 46	19: 21	33: 31	18: 27	63: 52	8.942^a^	0.063	
Age (y)	63.3 (8.1)	67.3 (10.1)	64.0 (6.6)	66.6 (7.5)	64.9 (8.6)	2.676	0.032	Nil
Education (y)	12.0 (2.8)	12.2 (2.9)	12.3 (3.2)	12.1 (3.1)	12.5 (2.9)	0.36	0.837	
Disease duration (y)	4.4 (3.0)	3.4 (2.1)	4.6 (2.2)	3.3 (2.2)	3.2 (1.8)	5.568	<0.001	bvFTD, SD > AD
ACE‐III total (100)	73.7 (16.7)	75.2 (15.3)	59.5 (16.6)	62.1 (16.2)	65.6 (17.1)	11.867	<0.001	PNFA, bvFTD > SD, LPA, AD
DAD IADL (100)	45.9 (27.0)	76.7 (25.8)	75.0 (21.4)	70.3 (26.9)	65.9 (26.4)	21.157	<0.001	all other groups > bvFTD
DAD BADL (100)	76.9 (22.4)	96 (9.0)	91.4 (11.5)	93.9 (11.5)	94 (10.9)	82.581	<0.001	all other groups > bvFTD
CBI‐R Abnormal Behaviour (100)	36.8 (23.9)	7 (10)	25 (24.8)	10.6 (17.5)	13 (14.1)	107.148^b^	<0.001	bvFTD > SD > PNFA, LPA, AD
CBI‐R Mood (100)	29.9 (21.9)	12.8 (12.4)	25.8 (21)	15.4 (16.3)	21.1 (17.6)	34.512^b^	<0.001	bvFTD > PNFA, LPA, AD; SD > PNFA
CBI‐R Eating Habits (100)	42.4 (28.8)	6.1 (10.9)	19.2 (20.4)	10.3 (17.5)	12.6 (16.6)	114.167^b^	<0.001	bvFTD > all other groups; SD > PNFA
CBI‐R Stereotypical and Motor (100)	46.5 (29.2)	14.3 (19.1)	40.9 (31)	13.9 (18.1)	21 (21.7)	90.142^b^	<0.001	bvFTD, SD > PNFA, LPA, AD
CBI‐R Motivation (100)	61.3 (30.9)	18.6 (19.5)	37.9 (30.3)	21 (23.6)	27.2 (24.4)	101.396^b^	<0.001	bvFTD > all other groups; SD > PNFA, LPA

*Notes*: Values are mean + standard deviation. Missing data: Education: 1 AD.

Abbreviations: ACE‐III, Addenbrooke's Cognitive Examination–Third edition; AD, Alzheimer's disease; bvFTD, behavioural variant frontotemporal dementia; CBI‐R, Cambridge Behavioural Inventory‐Revised; DAD BADL, basic activities of daily living on the Disability Assessment for Dementia; DAD IADL, instrumental activities of daily living; f, female; LPA, logopenic progressive aphasia (i.e., logopenic variant primary progressive aphasia); m, male; PNFA, progressive nonfluent aphasia (i.e., nonfluent variant primary progressive aphasia); SD, semantic dementia (i.e., semantic variant primary progressive aphasia); y, years.

^a^ = *χ* ^2^ test.

^b^ = Kruskal–Wallis analysis of variance.

On average, participants completed 2.4 clinical visits (range: 1–8 visits) and 3.5 carer assessments (range: 1–12 review assessments). Retention in the study varied across groups, with between 24% and 50% of participants completing 4 annual review assessments (Table S1).

Patients were excluded from the study if they were not proficient in English, did not have a reliable informant, or had a history of psychiatric illness, brain injury, or substance abuse. Patients initially diagnosed with bvFTD, PNFA, SD, LPA, or AD who subsequently developed another dementia disorder (e.g., Parkinson's plus syndrome, motor neuron disease, etc.) were also excluded to avoid contamination of functional capacity by the emergence of motor disturbances.

All participants or their person responsible (i.e., spouse, family member, etc.) provided written informed consent in accordance with the Declaration of Helsinki. The South Eastern Sydney Local Health District, University of New South Wales, and University of Sydney ethics committees approved the study (HREC 10/126).

### 2.2 Functional capacity

The Disability Assessment for Dementia (DAD)^14^ was used as the measure of functional ability. This informant‐based survey measures functional proficiency across 23 IADLs and 17 BADLs. The IADL domains include “meal preparation,” “telephoning,” “going on an outing,” “finance and correspondence,” “medications,” and “leisure and housework,” and the BADL domains include “hygiene,” “dressing,” “continence,” and “eating.” Questions that were not applicable to the patient were excluded to avoid activity bias (e.g., if the participant had never been involved in finance or cooking activities) and associated scores were adjusted accordingly. The IADL and BADL DAD scores were reported as a percentage of remaining ability, with lower scores representing poorer day‐to‐day functioning. The DAD was completed at each clinical visit with the assistance of an experienced psychologist (M.D'M., S.C.C.) or occupational therapist. When a patient was no longer able to attend the clinic at routine follow‐up, the carer was invited to complete the DAD remotely via telephone or a questionnaire version, until discontinuation.

### 2.3 General cognition

At each clinical visit, patients completed the Addenbrooke's Cognitive Examination‐III (ACE‐III).^24^ or ACE‐Revised (ACE‐R).^25^ The ACE provides an index of overall cognitive function across five cognitive domains (Attention and Orientation [/18], Memory [/26], Verbal Fluency [/14], Language [/26], and Visuospatial [/16]), summed to create an overall cognition score (/100). ACE‐R scores were converted to ACE‐III scores using a validated algorithm.^26^

### 2.4 Behavioural changes

Behavioural changes were assessed using the Cambridge Behavioural Inventory‐Revised (CBI‐R). The CBI‐R is an informant‐based questionnaire that evaluates behaviour, mood, cognition, and everyday skills.^27^ Carers rate the frequency of each item on a 5‐point scale, where 0 = never, 1 = a few times per month, 2 = a few times per week, 3 = daily occurrence, and 4 = constant occurrence. Data for five behavioural subdomains were used: abnormal behaviour (e.g., exhibiting socially embarrassing behaviour), mood (e.g., appearing sad or depressed), eating changes (e.g., restricted diet of specific foods), stereotypical behaviours (e.g., adhering to rigid daily routines), and motivation (e.g., appearing indifferent). Subdomain scores were converted to percentages to allow for domain comparison, with higher scores denoting greater behavioural change (i.e., worse outcomes). The CBI‐R was completed by the carer at each review assessment, either in‐person at the clinic or remotely, until discontinuation.

### 2.5 Statistical analyses

Data were analysed using IBM SPSS Statistics, 29.0 (SPSS Inc., Chicago, Ill., USA), and figures were created with SPSS and GraphPad Prism 9 (GraphPad Software, San Diego, CA, USA). The distribution normality of demographic, neuropsychological, and behavioural data was determined with Shapiro–Wilk tests.

Normally distributed baseline variables were compared across groups using one‐way analyses of variance (ANOVAs) followed by Bonferroni post hoc tests. Non‐normally distributed data or data with unequal variances were analysed using Kruskal–Wallis ANOVA followed by Dunn‐Bonferroni pairwise comparisons. A chi‐squared test (*χ* ^2^) was used to determine group differences for the categorical measure of sex. For all analyses, overall statistical significance was set at *p* < 0.05.

A linear mixed effects (LME) model was run to assess longitudinal changes in functional capacity across dementia groups. A combined linear mixed effects model (LME) was run to allow direct comparison between IADL and BADL variables. This model included fixed effects of diagnosis, index (i.e., IADL, BADL), follow‐up time in years, and the interaction between diagnosis and follow‐up time. A random intercept was included to account for individual variability associated with each patient at baseline. Index, diagnosis, follow‐up time in years, the interaction between index and diagnosis, and the interaction between index, diagnosis, and follow‐up time were analysed. Null hypotheses of no change in all fixed effects were tested. To adjust for multiple *post hoc* group comparisons of slopes, the *p*‐value of these comparisons was set at 0.01.

Finally, associations between baseline assessment DAD IADL and DAD BADL raw performance scores and the ACE‐III total and CBI‐R subdomain scores were investigated using Spearman's rank‐order correlations. Bootstrapped bias corrected 95% confidence intervals (CIs) were reported. Evidence of correlations were reported when both lower and upper CI values were greater than zero.

## 3 RESULTS

### 3.1 Baseline assessment

#### 3.1.1 Demographic and clinical profiles at baseline assessment

No significant differences were observed between patient groups with regard to age, sex, and years of education (Table 1). Disease duration, however, was significantly longer in the bvFTD and SD groups relative to the AD group (both *p*‐values ≤ 0.005).

#### 3.1.2 Functional capacity

The bvFTD group was rated as displaying worse functional outcomes at baseline on both DAD measures (IADL, BADL) relative to the other groups (all *p*‐values < 0.001) (Table 1). No other significant between group differences were observed (all *p*‐values ≥ 0.212).

#### 3.1.3 Cognitive performance

Baseline performance on the ACE‐III total was found to differ significantly across groups (*p* < 0.001). This was driven by lower performance in the SD, LPA, and AD groups compared to the PNFA and bvFTD groups (all *p*‐values ≤ 0.018) (Table 1), with no other significant differences present (all *p*‐values ≥ 0.177). The analyses on ACE‐III subdomain baseline performance across groups are presented in the Supplementary Material Results and Table S2.

#### 3.1.4 Behavioural changes

Significant group differences emerged for carer‐rated behavioural changes on the CBI‐R at baseline (Table 1). Overall, bvFTD patients were reported as displaying more behavioural and mood disturbances than other groups. Similarly, the SD group was rated as displaying greater behavioural and mood changes relative to the PNFA, LPA, and AD groups (see Supplementary Material for full details).

### 3.2 Longitudinal functional decline

**FIGURE 1:**
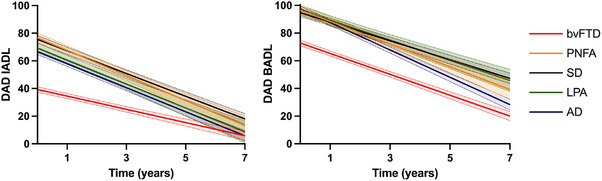
Predicted Disability Assessment for Dementia (DAD) instrumental activities of daily living (IADL), and DAD basic activities of daily living (BADL) scores over time. Note: Values are means and standard error of the mean from the linear mixed effects model.

Figure 1 displays the functional decline trajectories (measured by DAD IADL and DAD BADL) across groups over the study period (7 years post‐baseline) (see also Figures S1 and S2). Significant main effects were observed for index, diagnosis, and time (all *p*‐values < 0.001; Table S3). Specifically, the cohort showed more impairment in IADL than BADL (irrespective of diagnosis or time), and functional capacity declined significantly over time (irrespective of diagnosis or index). Importantly, a significant main effect of diagnosis indicated that the decline in overall functional capacity (both IADL and BADL) was more severe in the bvFTD group (reference group) compared to other groups (all *p*‐values < 0.001). A significant two‐way interaction (index × diagnosis) indicated differences between groups in IADL and BADL measures, regardless of time.

Importantly, a significant three‐way interaction (index × diagnosis × time) was observed (*p* = 0.001), indicating that the overall progression (i.e., slope) varied across DAD measures and across groups (Tables S4 and S5). Relative to the bvFTD group, the other clinical groups demonstrated increasing IADL impairment with disease progression (IADL decline was between 69% and 83% greater than bvFTD; mean annual decline of IADL: bvFTD: −4.9, PNFA: −9.1, SD: −8.2, LPA: −8.8, AD: −8.9). The rates of BADL decline across groups were more variable, with AD displaying the greatest decline (mean annual decline on BADL: −10.1), SD demonstrating the least decline (−6.8), and bvFTD, PNFA, and LPA positioned in‐between these two extremes (bvFTD: −7.6, PNFA: −8.2, LPA: −7.1).

### 3.3 Correlations between the DAD IADL and BADL, and ACE‐III total and CBI‐R subdomain scores at baseline assessment in each group

**FIGURE 2:**
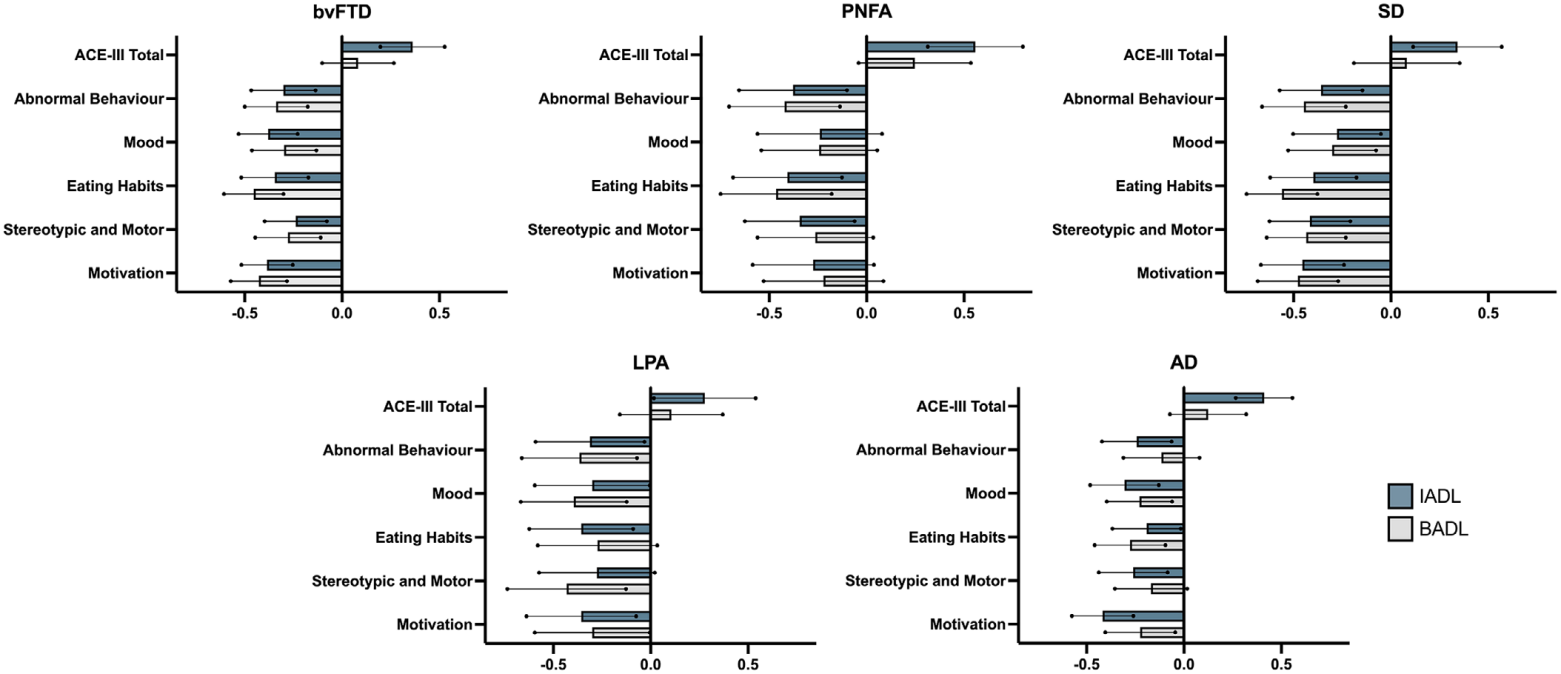
Spearman's rank‐order correlations between Disability Assessment for Dementia (DAD) instrumental activities of daily living (IADLs) and basic activities of daily living (BADLs) and the Addenbrooke's Cognitive Examination–Third edition (ACE‐III) total and Cambridge Behavioural Inventory‐Revised (CBI‐R) subdomain measures at baseline in each group. Floating lines represent the 95% confidence intervals (CIs). Evidence of correlation is demonstrated when both lower and upper CI values do not include zero. Note that the lower ACE‐III scores indicate worse cognition, while higher CBI‐R scores reflect greater behavioural disturbance.

Correlation confidence intervals are displayed in Figure 2 and reported in Table S6. For all groups, overall cognition (i.e., ACE‐III total) correlated with high‐level instrumental (i.e., IADL) but not with basic (i.e., BADL) activities of daily living. Correlations between ADL performance and behavioural and mood changes revealed distinct profiles across groups. For the bvFTD and SD groups, IADL and BADL deficits were associated with disturbances in all CBI‐R subdomains. In contrast, for the PNFA, LPA, and amnestic AD groups, changes in CBI‐R scores were predominantly related to IADL performance. Relations between CBI‐R scores and BADL across groups were more variable.

## 4 DISCUSSION

Disturbances in functional capacity are well‐established in dementia; however, longitudinal studies evaluating their evolution over the disease course are lacking. This study aimed to analyse the evolving functional profiles in a well‐characterised cohort of patients with FTD, as well as in amnestic and language presentations of AD. Overall, our findings revealed significantly greater instrumental and basic functional impairment in bvFTD relative to the other patient groups at baseline and over the study period. In contrast, the other dementia syndromes showed similar profiles characterised by mild to moderate deficits at baseline assessment but a faster decline over time. The functional profiles of these dementia syndromes occurred in the context of distinct patterns of associations with behaviour and cognitive changes. Our findings highlight the distinct and evolving profiles of the various FTD and AD syndromes and offer valuable insights to enhance disease management and psychoeducation.

Our investigations revealed distinct longitudinal profiles, which varied according to the DAD subscale and dementia subtype. For IADLs, the bvFTD group was significantly more impaired than the other groups at the initial assessment and over the study period. Inspection of the slope of change (i.e., trajectories relative to baseline), however, indicated that the other dementia groups experienced an annual decline that was almost twice that observed in the bvFTD group (∼9% vs. 4.8%), likely attributable to their greater functional impairment at baseline. By 7 years post‐baseline, the PNFA, SD, LPA, and AD groups had largely approached the projected level of IADL impairment in the bvFTD group (indicating severe difficulties with complex activities such as meal preparation, adherence to medication, managing finances, etc.). For BADLs, bvFTD again experienced the greatest impairments at baseline compared to the other groups. Progression (i.e., slope) of BADL deficits over time, however, was variable across the groups and was most pronounced in AD and least pronounced in SD (∼10% vs. ∼7% annual decline, respectively). Overall, these findings corroborate reports of early and disproportionate deficits in all aspects of functional capacity in bvFTD compared to other forms of dementia.^4^, ^5^, ^6^, ^10^, ^13^, ^15^, ^16^ These functional changes in bvFTD have been associated with higher rates of carer distress and burden^28^, ^29^ and have been found to prompt earlier engagement with at‐home services, including allied health support and respite care, relative to other dementia subtypes.^29^, ^30^

A second objective of this study was to determine the relations between functional changes and cognition and behaviour in these dementia syndromes. In all groups, general cognition was positively related to IADLs but not BADLs. In other words, the ability to perform high‐level tasks (e.g., shopping, finance, etc.) was affected by the severity of the cognitive deficits, regardless of the dementia diagnosis. In contrast, the ability to perform basic activities (e.g., bathing and dressing) was not related to cognitive capacity. These findings demonstrate the presence of different mechanisms supporting IADL and BADL proficiency, with the former relying, at least in part, on preserved cognitive functioning, whereas the latter does not. Further, these results support the view that bvFTD patients experience widespread impairments encompassing both BADLs and IADLs at clinical presentation, despite cognition remaining less compromised compared with the other clinical groups.^5^

Turning our attention to behaviour, our investigations revealed significant associations between IADLs or BADLs and all behaviour categories surveyed (abnormal behaviour, mood, eating habits, stereotypical behaviour, and motivation) in bvFTD and SD. In contrast, for the other dementia syndromes, a distinction between BADLs and IADLs emerged concerning their relations between functional capacity and behaviour, similar to that observed with general cognition. Indeed, significant associations were found between IADLs and all behaviour categories in PNFA, LPA, and AD (with the exception of stereotypic behaviour in LPA, and mood and motivation in PNFA). In contrast, correlations between BADLs and behaviour were less consistent across these clinical groups. In PNFA, BADLs were significantly associated with abnormal behaviour and with eating habits only. For LPA, however, all behaviour categories, with the exception of eating habits, significantly correlated with BADLs. Finally, in AD, BADLs were significantly associated with mood, eating habits, and motivation. Overall, for both IADLs and BADLs, the strengths of the significant correlations with behaviour were more variable in PNFA than in the other clinical groups.

Another important finding to highlight is the marked behavioural disturbances observed in SD at baseline. Although not as severe as those found in bvFTD, significantly higher levels of behaviour changes across all categories measured were present in SD relative to LPA, PNFA, and AD. Our findings align well with previous reports^7^, ^9^, ^16^, ^21^, ^23^, ^31^ and support the need to rethink how we conceptualise this syndrome.^22^, ^23^ While semantic loss is the core feature of SD, changes in other domains, including behaviour, are common.^32^ Supporting carers to anticipate and navigate these behavioural symptoms while enabling the person with SD to retain some independence given their relatively milder changes in basic activities of daily living, will be a crucial next step.

Finally, in the remaining clinical groups (PNFA, LPA, and AD), ADL changes (instrumental and basic) and behaviour were of similar severity at baseline, despite PNFA demonstrating better cognition than the other two groups. It is important to note that while the functional decline profiles of PNFA and LPA were largely comparable in our study, some evidence suggests that LPA patients may exhibit more pronounced declines in IADLs compared to PNFA patients over time.^9^, ^12^, ^16^, ^33^ Differences between measurement tools, such as the utilisation of binary (e.g., “able/not able” in the DAD) versus graded (e.g., “never, sometimes, etc.” in the CBI‐R) scales could account for these apparent disparities.

Taken together, our results have important clinical implications. Indeed, these findings suggest that interventions targeting IADLs will be most successful by focusing on a combination of cognitive and behavioural approaches. In contrast, if BADLs are the focus, addressing aspects of behaviour may be sufficient. This information will be important for carers and clinicians as they work to understand and develop effective strategies to manage functional deficits.

Several methodological issues warrant attention. First, our scores for assessing functional, behavioural, and mood changes were derived from a composite of related but distinct informant questions. Future research is needed to ascertain how individual items within each domain/instrument contribute to these overall scores. Second, this study relied on informant‐based questionnaires to evaluate the changes in behaviour and mood. While such surveys are useful and cost‐effective tools for evaluating daily life changes, they are not without limitations. Carers may over‐ or under‐report symptom frequency or severity based on various variables such as interpretation of questions, premorbid personality traits, and current mood or levels of engagement in daily activities.^34^, ^35^, ^36^ Integration of performance‐based measures with informant reports will provide a more comprehensive understanding of the behavioural changes in dementia yet may prove increasingly difficult to implement in the advanced disease stages. Third, given the extensive study period, retention rates varied among the groups, falling below 50% for all groups at 4 years post‐baseline assessment. High attrition rates were observed in PNFA, LPA, and AD participants, which may have influenced the data modeling. Fourth, in this instance, linear modeling was the most appropriate method to compare the functional profiles across groups; however, we acknowledge that functional decline in some groups likely occur in a non‐linear fashion. As such, future research may wish to investigate whether other modeling methods better capture the complexities of real‐life disease progression in these disease groups. Finally, our study excluded individuals who went on to develop a Parkinson's plus and/or frank motor syndrome (e.g., amyotrophic lateral sclerosis). Individuals who develop motor features will experience additional challenges with self‐care and everyday tasks.^37^, ^38^ Accordingly, our findings are mainly relevant to patients without marked motor features. Further research will be needed to compare the evolving functional, cognitive, and behavioural changes in patients with and without motor features.

In summary, this study is one of the largest and most comprehensive to date in demonstrating the distinct functional profiles across FTD and AD syndromes with disease progression. Our findings also identify how functional capacity profiles at baseline assessment relate to cognition and behaviour among syndromes. These findings have important implications for clinical profiling, management, and psychoeducation.

## AUTHOR CONTRIBUTIONS

David Foxe, Muireann Irish, and Olivier Piguet contributed to the design and conceptualisation of the study, analysis and interpretation of data, and drafting of the manuscript. James Muggleton contributed to analysing and interpreting the data, improving the study design, and revising the manuscript. Sau Chi Cheung, Mirelle D'Mello, Yun Tae Hwang, and Nicholas J. Cordato contributed to the acquisition and interpretation of data and revising the manuscript.

## CONFLICT OF INTEREST STATEMENT

The authors declare no conflicts of interest. Author disclosures are available in the Supporting Information.

## Supporting information



Supporting Information

Supporting Information

## Data Availability

The datasets generated during and/or analysed during the current study are available from the corresponding author on reasonable request. No part of the study procedures or analyses were preregistered prior to the research being undertaken. The Addenbrooke's Cognitive Examination‐Third edition (ACE‐III) is freely available at https://frontierftd.org (accessed on May 31 2024). Legal copyright restrictions prevent public archiving of the other tests used in this research. These materials can be obtained from the copyright holders in the cited references.
