# Supplementary material for: Longitudinal changes in functional capacity in frontotemporal dementia and Alzheimer's disease
Source: Alzheimers Dement (Amst). 2024 Nov 15;16(4):e70028. doi: 10.1002/dad2.70028 (PMC11567831; doi:10.1002/dad2.70028)
Supplement: Supplementary file 1 — Supporting Information [file DAD2-16-e70028-s002.pdf]

Supplementary Material

Longitudinal changes in functional capacity frontotemporal dementia and Alzheimer's disease

David Foxe*, Muireann Irish, Sau Chi Cheung, Mirelle D'Mello, Yun Tae Hwang, James Muggleton, Nicholas J. Cordato, Olivier Piguet

***Corresponding author:** David Foxe, Brain and Mind Centre, The University of Sydney, 94 Mallett St, Sydney NSW 2006, Australia

Email: david.foxe@sydney.edu.au

RESULTS

Baseline demographics, ACE-III and DAD

Baseline ACE-III subdomains

Overall group differences were present for all baseline ACE-III subdomain raw scores (all p values < .001). Post hoc tests revealed cognitive profiles characteristic of each dementia subtype (Supplementary Table 2). Specifically, LPA and AD group performances were comparable (p = 1.000) on Attention, and more impaired than the bvFTD, SD and PNFA groups (all p values $\leq$ .026). On the (verbal) Memory subdomain, SD, AD, and LPA group performances were comparable (all p values = 1.000) and more impaired than PNFA and bvFTD (all p values $\leq$ .013). The SD and LPA groups were more impaired than the AD group on Fluency, while differences between PNFA and AD on this measure did not reach significance (p = .055). Fluency performances did not differ between SD, LPA, PNFA and bvFTD groups (all p values > .119). On the Language subdomain, the LPA group were more impaired than the AD and bvFTD groups (both p values < .017) but not the PNFA group (p = .371). The SD group were the most impaired on Language (vs. all other groups: all p values < .001). Lastly, the AD

and LPA groups were comparable (p = 1.000) and disproportionately impaired on the Visuospatial submain, relative to the other groups (AD vs all other groups, *p* values < .001; LPA vs. PNFA and SD, *p* values ≤ .026; LPA vs. bvFTD, *p* = .176).

Baseline CBI-R subdomains

Direct comparisons of the patient groups on the baseline CBI-R subdomain raw scores are presented in Table 1. Overall, carers of the bvFTD group endorsed more Abnormal Behaviours, Eating changes, and Motivational disturbance (i.e., less motivation) than all other groups (all *p* values ≤ .004). BvFTD carers also reported more Mood disturbance and Stereotypical and Motor Behaviours than the PNFA, LPA and AD groups (all *p* values ≤ .021). Carers of the SD group endorsed more Abnormal Behaviours, and Stereotypic and Motor changes than the PNFA, LPA, and AD groups (all *p* values ≤ .039), and more Mood disturbance than the PNFA group (*p* = .011). No significant differences were reported between PNFA, LPA and AD on these domains (all *p* values ≥ .112).

Supplementary Table 1. Number of completed DAD assessments represented as a percentage of total group. DAD assessments were completed at each annual visit to the clinic or by telephone and/or questionnaire surveys.

Completed DADs	bvFTD	PNFA	SD	LPA	AD
1	100%	100%	100%	100%	100%
2	81%	74%	95%	67%	59%
3	52%	40%	67%	44%	39%
4	29%	31%	52%	24%	24%
5	15%	12%	25%	7%	13%
6	9%	7%	20%	4%	3%
7	5%	2%	10%	2%	3%

.

Supplementary Table 2. Cognitive profiles of the patient groups at baseline assessment

	bvFTD	PNFA	SD	LPA	AD	F	*p*	Post hoc test (Bonferroni corrected)
ACE-III Attention (18)	14.7 (3.2)	15.2 (2.8)	15.1 (2.6)	12.6 (3.7)	12.9 (3.7)	9.667	< .001	LPA, AD < bvFTD, SD, PNFA
ACE-III Memory (26)	17.7 (5.9)	20.3 (6.4)	12.9 (5.8)	14.4 (6)	13.1 (5.7)	19.284	< .001	SD, AD, LPA < PNFA, bvFTD
ACE-III Fluency (14)	6.2 (3.8)	5.2 (3.4)	5 (3.6)	4.7 (2.9)	7 (3.3)	5.879	< .001	LPA, SD < AD
ACE-III Language (26)	21.4 (4.9)	20.3 (4)	12.7 (5.1)	18.1 (5.4)	20.8 (4.8)	39.212	< .001	SD < all other groups; LPA < AD, bvFTD
ACE-III Visuospatial (16)	13.8 (2.6)	14.5 (1.6)	14.3 (2.2)	12.6 (2.7)	12.1 (3.5)	11.039	< .001	AD < bvFTD, SD, PNFA; LPA < SD, PNFA

Notes: Values are mean ± standard deviation.

Abbreviations: ACE-III: Addenbrooke's Cognitive Examination–Third edition; bvFTD: behavioural variant frontotemporal dementia; LPA: logopenic progressive aphasia (i.e., logopenic variant primary progressive aphasia); PNFA: progressive non-fluent aphasia (i.e., non-fluent variant primary progressive aphasia); SD: semantic dementia (i.e., semantic variant primary progressive aphasia).

Missing data: CBI-R: 1 bvFTD, 1 SD.

Supplementary Table 3. Linear mixed effects model of longitudinal changes on the DAD IADL and BADL: Type III Tests of Fixed Effects

	Numerator df	Denominator df	F	*p*-value
Intercept	1	465.243	4529.761	< .001
Index (i.e., IADL, BADL)	1	1929.361	552.090	< .001
Diagnosis	4	461.632	39.322	< .001
Follow-up time	1	2182.500	711.850	< .001
Index x Diagnosis	4	1929.570	8.611	< .001
Index x Diagnosis x Follow-up time	9	2041.685	6.148	< .001

Supplementary Table 4. Predicted DAD IADL, and DAD BADL scores over time in all dementia groups

DAD IADL

Time (years)	bvFTD		PNFA		SD		LPA		AD	
	Mean	S.E.M	Mean	S.E.M	Mean	S.E.M	Mean	S.E.M	Mean	S.E.M
0	39.31	2.00	76.66	3.64	75.53	2.77	69.25	3.50	66.71	2.17
1	34.53	1.87	67.60	3.35	67.32	2.61	60.60	3.24	58.01	2.04
2	29.75	1.87	58.54	3.40	59.10	2.57	51.95	3.44	49.31	2.08
3	24.97	1.99	49.47	3.77	50.89	2.65	43.31	4.03	40.61	2.28
6	10.64	2.91	22.28	6.01	26.24	3.47	17.37	6.93	14.51	3.45
7	5.86	3.31	13.22	6.94	18.03	3.86	8.72	8.05	5.81	3.93

DAD BADL

Time (years)	bvFTD		PNFA		SD		LPA		AD	
	Mean	S.E.M	Mean	S.E.M	Mean	S.E.M	Mean	S.E.M	Mean	S.E.M
0	72.83	2.00	96.62	3.64	94.77	2.77	96.63	3.50	97.44	2.17
1	65.29	1.87	88.43	3.35	87.99	2.61	89.37	3.24	87.57	2.04
2	57.75	1.86	80.24	3.40	81.20	2.57	82.11	3.44	77.70	2.08
3	50.21	1.99	72.05	3.77	74.42	2.65	74.85	4.03	67.83	2.28
6	27.59	2.91	47.47	6.01	54.06	3.47	53.07	6.93	38.23	3.45
7	20.05	3.31	39.28	6.94	47.27	3.86	45.81	8.05	28.36	3.93

Notes, values are derived from the linear mixed effects model. Values are the mean and standard error of the mean.

Supplementary Table 5. Predicted annual rates of change on the DAD IADL, and DAD BADL

		Annual rate of change	S.E.M.
**DAD IADL**	bvFTD	-4.87	0.77
	PNFA	-9.07	0.77
	SD	-8.24	1.23
	LPA	-8.56	1.23
	AD	-8.92	0.81
		**Annual rate of change**	**S.E.M.**
**DAD BADL**	bvFTD	-7.62	0.81
	PNFA	-8.20	1.37
	SD	-6.81	1.37
	LPA	-7.18	0.69
	AD	-10.08	0.59

Notes, values are derived from the linear mixed effects models. Values are the mean and standard error of the mean.

Supplementary Table 6. Spearman's rank-order correlations and 95% CI between DAD IADL, DAD BADL, and ACE-III Total and CBI-R subdomain scores at baseline.

		IADL	BADL
**bvFTD**	ACE-III Total	**.377 (.527 – .197)**	0.094 (-.102 – .266)
	Abnormal Behaviour	**-.310 (-.136 – -.466)**	**-.345 (-.500 – -.176)**
	Mood	**-.386 (-.228 – -.531)**	**-.299 (-.463 – -.132)**
	Eating Habits	**-.242 (-.173 – -.518)**	**-.462 (-.606 – -.301)**
	Stereotypic and Motor	**-.361 (-.078 – -.397)**	**-.283 (-.446 – -.110)**
	Motivation	**-.390 (-.253 – -.517)**	**-.430 (-.571 – -.283)**
		IADL	BADL
**PNFA**	ACE-III Total	**.603 (.314 – .802)**	.271 (-.041 – .535)
	Abnormal Behaviour	**-.407 (-.655 – -.101)**	**-.454 (-.706 – -.137)**
	Mood	-.250 (-.560 – .079)	-.244 (-.541 – .054)
	Eating Habits	**-.461 (-.686 – -.127)**	**-.504 (-.750 – -.180)**
	Stereotypic and Motor	**-.370 (-.625 – -.062)**	-.282 (-.560 – .034)
	Motivation	-.295 (-.585 – .037)	-.251 (-.529 – .086)
		IADL	BADL
**SD**	ACE-III Total	**.365 (.114 – .569)**	.094 (-.190 – .353)
	Abnormal Behaviour	**-.381 (-.571 – -.146)**	**-.461 (-.662 – -.232)**
	Mood	**-.277 (-.502 – -.052)**	**-.310 (-.528 – -.076)**
	Eating Habits	**-.420 (-.620 – -.177)**	**-.581 (-.741 – -.378)**

	Stereotypic and Motor	**-.433 (-.623 – -.209)**	**-.449 (-.638 – -.232)**
	Motivation	**-.485 (-.668 – -.241)**	**-.490 (-.684 – -.271)**
		IADL	BADL
**LPA**	ACE-III Total	**.300 (.017 – .539)**	.117 (-.158 – .370)
	Abnormal Behaviour	**-.341 (-.591 – -.032)**	**-.398 (-.662 – -.070)**
	Mood	**-.330 (-.595 – -.005)**	**-.412 (-.667 – -.123)**
	Eating Habits	**-.387 (-.623 – -.090)**	-.281 (-.580 – .0340)
	Stereotypic and Motor	-.304 (-.573 – .021)	**-.466 (-.736 – -.127)**
	Motivation	**-.397 (-.638 – -.075)**	**-.322 (-.595 – -.005)**
		IADL	BADL
**AD**	ACE-III Total	**.425 (.266 – .557)**	.128 (-.072 – 0.320)
	Abnormal Behaviour	**-.247 (-.421 – -.064)**	-.113 (-.311 – .080)
	Mood	**-.302 (-.482 – -.129)**	**-.231 (-.396 – -.061)**
	Eating Habits	**-.199 (-.367 – -.017)**	**-.277 (-.459 – -.095)**
	Stereotypic and Motor	**-.261 (-.437 – -.084)**	-.169 (-.355 – .017)
	Motivation	**-.435 (-.576 – -.260)**	**-.236 (-.404 – -.045)**

Note: Evidence of correlation is demonstrated when both lower and upper CI values do not include 0 (in bold).

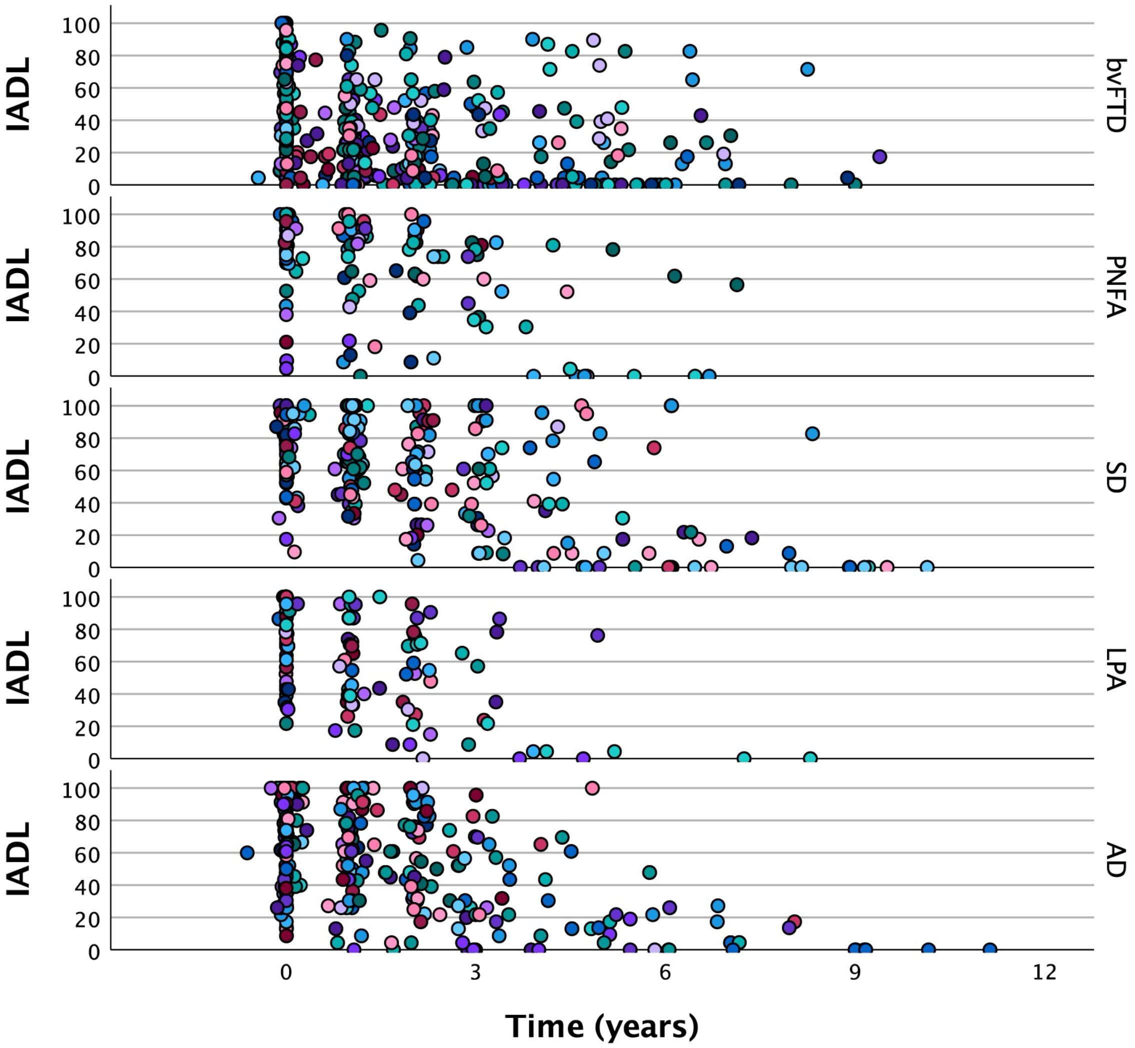

Supplementary Figure 1. Individual scatterplots of instrumental activities of daily living (IADL) scores on the Disability Assessment for Dementia (DAD) across groups over time. The IADL DAD scores are shown as a percentage of remaining ability, with lower scores indicating worse day-to-day functioning. Each colour represents a different individual.

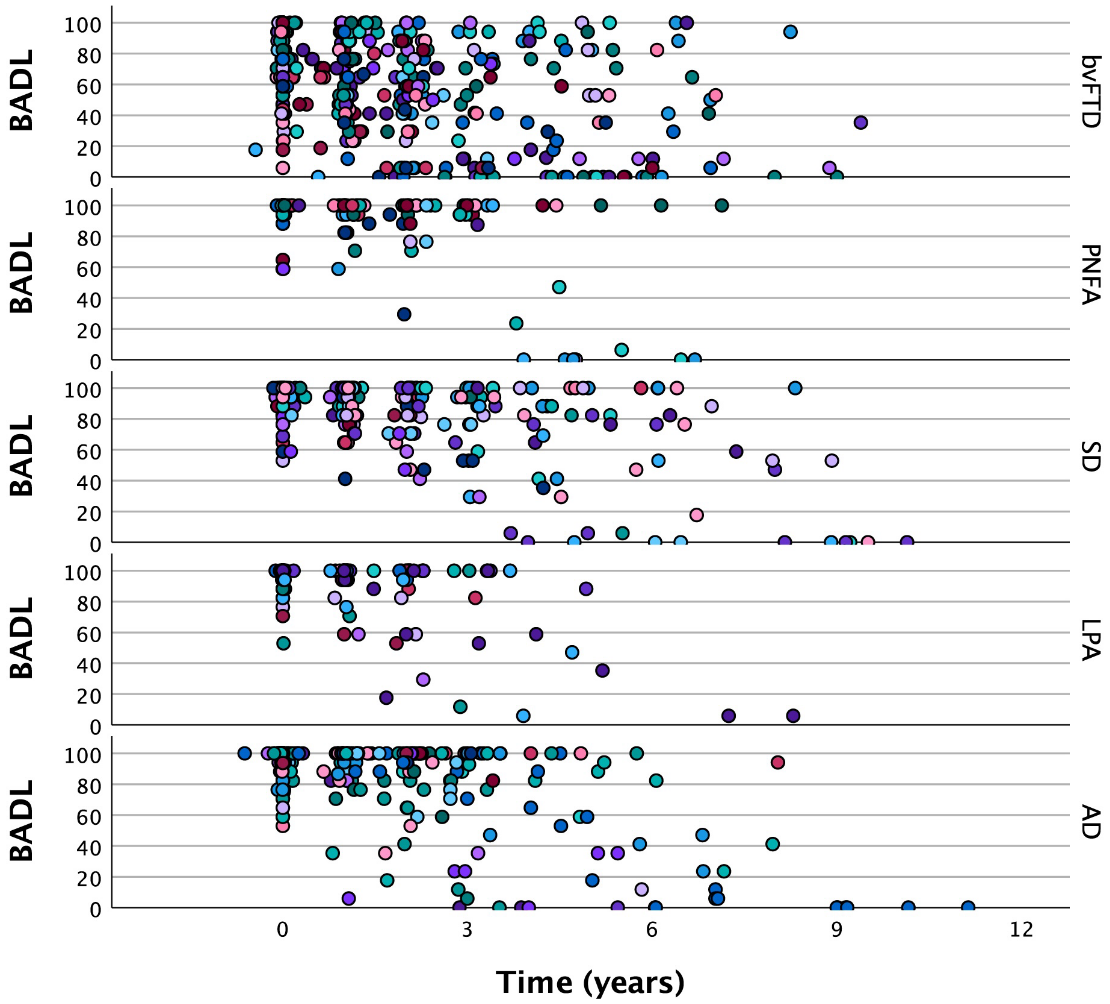

Supplementary Figure 2. Individual scatterplots of basic activities of daily living (BADL) scores on the Disability Assessment for Dementia (DAD) across groups over time. The BADL DAD scores are presented as a percentage of remaining ability, with lower scores indicating worse day-to-day functioning. Each colour represents a different individual.
